# The effect of DNA extraction methodology on gut microbiota research applications

**DOI:** 10.1186/s13104-016-2171-7

**Published:** 2016-07-26

**Authors:** Konstantinos Gerasimidis, Martin Bertz, Christopher Quince, Katja Brunner, Alanna Bruce, Emilie Combet, Szymon Calus, Nick Loman, Umer Zeeshan Ijaz

**Affiliations:** 1 Human Nutrition, School of Medicine, College of Medical, Veterinary and Life Sciences, Glasgow Royal Infirmary, University of Glasgow, Glasgow, UK; 2 Warwick Medical School, University of Warwick, Warwick, UK; 3 School of Engineering, University of Glasgow, Oakfield Avenue, Glasgow, G12 8LT UK; 4 Institute of Microbiology and Infection, University of Birmingham, Birmingham, B15 2TT UK

**Keywords:** Metagenomics, DNA extraction, Benchmarking, Diversity, PCR, 16S rRNA gene

## Abstract

**Background:**

The effect that traditional and modern DNA extraction methods have on applications to study the role of gut microbiota in health and disease is a topic of current interest. Genomic DNA was extracted from three faecal samples and one probiotic capsule using three popular methods; chaotropic (CHAO) method, phenol/chloroform (PHEC) extraction, proprietary kit (QIAG). The performance of each of these methods on DNA yield and quality, microbiota composition using quantitative PCR, deep sequencing of the 16S rRNA gene, and sequencing analysis pipeline was evaluated.

**Results:**

The CHAO yielded the highest and the QIAG kit the lowest amount of double-stranded DNA, but the purity of isolated nucleic acids was better for the latter method. The CHAO method yielded a higher concentration of bacterial taxa per mass (g) of faeces. Sequencing coverage was higher in CHAO method but a higher proportion of the initial sequencing reads were retained for assignments to operational taxonomic unit (OTU) in the QIAG kit compared to the other methods. The QIAG kit appeared to have longer trimmed reads and shorter regions of worse quality than the other two methods. A distinct separation of α-diversity indices between different DNA extraction methods was not observed. When compositional dissimilarities between samples were explored, a strong separation was observed according to sample type. The effect of the extraction method was either marginal (Bray–Curtis distance) or none (unweighted Unifrac distance). Taxon membership and abundance in each sample was independent of the DNA extraction method used.

**Conclusions:**

We have benchmarked several DNA extraction methods commonly used in gut microbiota research and their differences depended on the downstream applications intended for use. Caution should be paid when the intention is to pool and analyse samples or data from studies which have used different DNA extraction methods.

**Electronic supplementary material:**

The online version of this article (doi:10.1186/s13104-016-2171-7) contains supplementary material, which is available to authorized users.

## Background

The introduction of molecular biology techniques and deep sequencing in microbiology has revolutionised our interest and advanced our understanding on the role of gut microbiota in health and disease. Isolation and purification of bacterial genomic DNA from gut mucosal and luminal contents is a crucial initial step to ensure a high yield and quality of isolated nucleic acids, and unbiased representation of microbial communities. Over the past decade, several proprietary DNA extraction kits have been developed and became commercially available with the intention to replace the more laborious, time consuming original approaches [1, 2]. There is good evidence showing that different DNA extraction kits will generate different results in terms of: amount and quality of extracted DNA, inhibitors of PCR reactions, and influences on bacterial community composition [1–5]. The effects that various DNA extraction methods may have on traditional downstream methods (e.g. quantitative PCR) compared to modern next generation sequencing approaches have not been extensively explored [1, 2]. Hereby, we have performed a benchmarking study and explored the effect of three popular faecal DNA extraction methods on yield and quality of isolated DNA, as well as microbiota composition determined by a typical sequencing analysis pipeline, using traditional molecular microbiology techniques and high-throughput next generation sequencing.

## Results

### Effect on yield and purity of isolated DNA

**Fig. 1:**
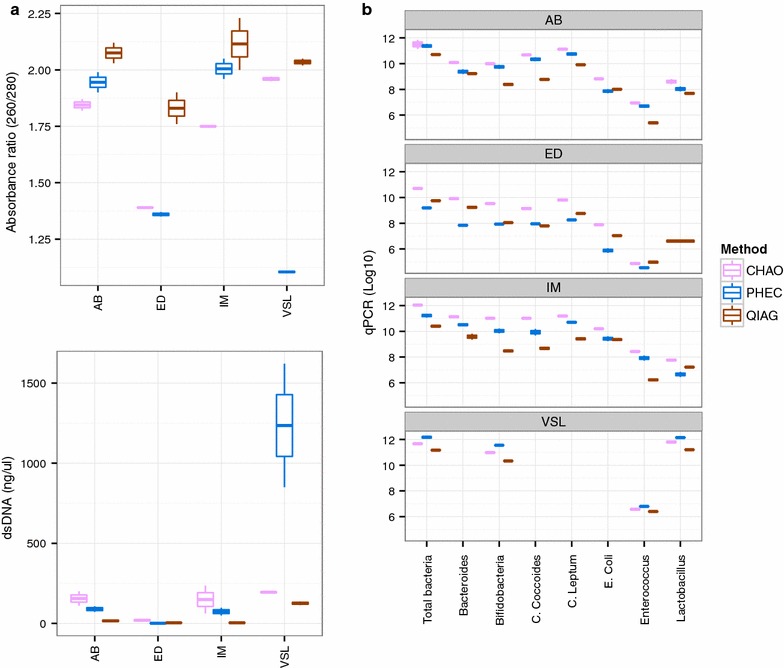
Effect of DNA extraction method on double stranded DNA yield, nucleic acids quality metrics and quantitative PCR analysis. The CHAO method gave the maximum concentration of amplicons per mass (g) of faecal samples (**a**). While PHEC method had the highest double stranded DNA concentration (*bottom left*) for the probiotic capsule, the absorbance ratio 260/280 indicated that the extracted nucleic acids were of low purity (*top left*). *Right panel* (**b**) shows the 16S rRNA amplicon copies in qPCR analysis. ED, AB, IM correspond to the three faecal sample and VSL to the proprietary probiotic capsule ID respectively

Beginning with the same faecal material mass, the chaotropic method (CHAO) produced the highest and the Qiagen kit (QIAG) the lowest yield of double stranded DNA (Fig. 1a). However, for the absorbance ratio at 260/280 nm, a metric of isolated nucleic acids purity, the QIAG kit performed best and the CHAO method worst (Fig. 1a). Extraction of a proprietary probiotic capsule with the phenol/chloroform (PHEC) method gave the highest yield of double-stranded DNA but this was comparatively of very poor quality.

### Effect on quantitative estimates of major bacterial taxa

Independently of faecal sample or bacterial taxon explored, the same mass of faecal sample gave a higher concentration (per g of original faeces) of 16S rRNA amplicon copies with the CHAO method in qPCR analysis (Fig. 1b). For the probiotic capsule, the PHEC method produced the highest concentration of amplicon copies for all taxa identified.

### Effect on microbial community determined through 16S rRNA amplicon sequencing

**Fig. 2:**
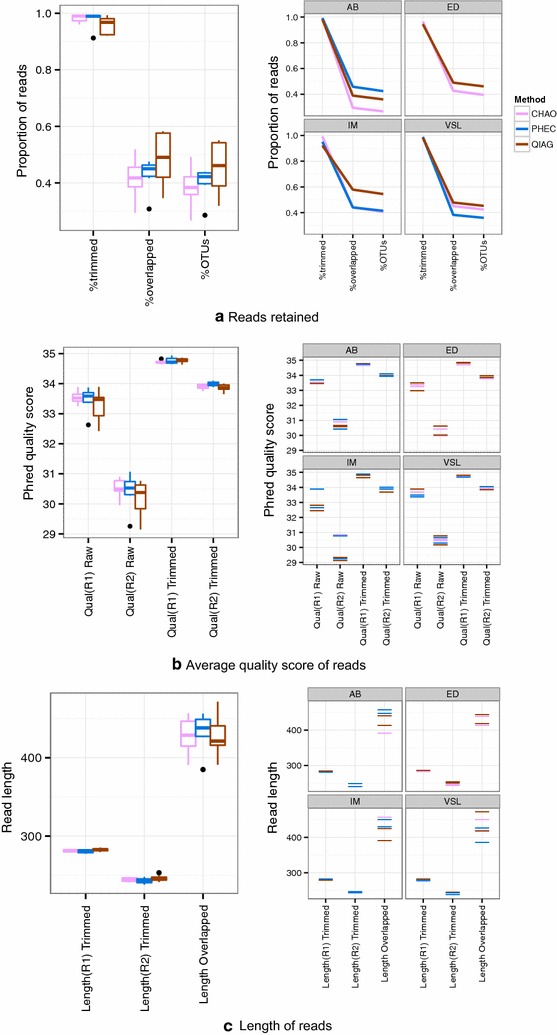
Effect of DNA method on each step of typical bioinformatics analysis pipeline. The *left* and *right columns* show organisation by per method and per sample basis, respectively. In terms of the total useful reads that are mapped to OTU, QIAG kit performed better than the other two methods as roughly 50 % of the reads were mapped to OTUs during the construction stage (see Additional file 2 : Table S1 for actual read numbers and Additional file 1 : Figure S1 summary statistics). In terms of quality trimming, QIAG lost more reads than the other two methods. ED, AB, IM correspond to the three faecal sample and VSL to the proprietary probiotic capsule ID respectively

The mean (SD) number of reads per method was higher for the CHAO and PHEC than the QIAC method (Additional file 1: Figure S1, Additional file 2: Table S1). In terms of sequencing yield, more reads from the QIAG kit were obtained for OTU construction (Fig. 2a). In accordance to our recent publication, the reverse reads for the Illumina platform were of worse quality than the forward ones [6] and hence the difference in the number of reads after quality trimming. Trimming improved the read quality to an extent and sequencing reads originated from the PHEC method had better quality than the other two approaches (Fig. 2b). In terms of read length, the QIAG kit appeared to have longer trimmed reads and shorter regions of worse quality, than the other two methods. The PHEC method produced the longest overlapped reads (Fig. 2c).

### Effect on 16S rRNA amplicon sequencing based community composition

**Fig. 3:**
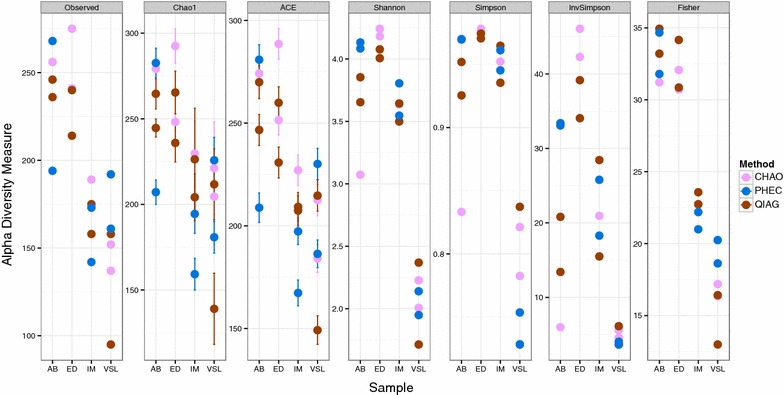
The effect of the DNA extraction methods on various microbial α-diversity community estimates. ED, AB, IM correspond to the three faecal sample and VSL to the proprietary probiotic capsule ID respectively

**Fig. 4:**
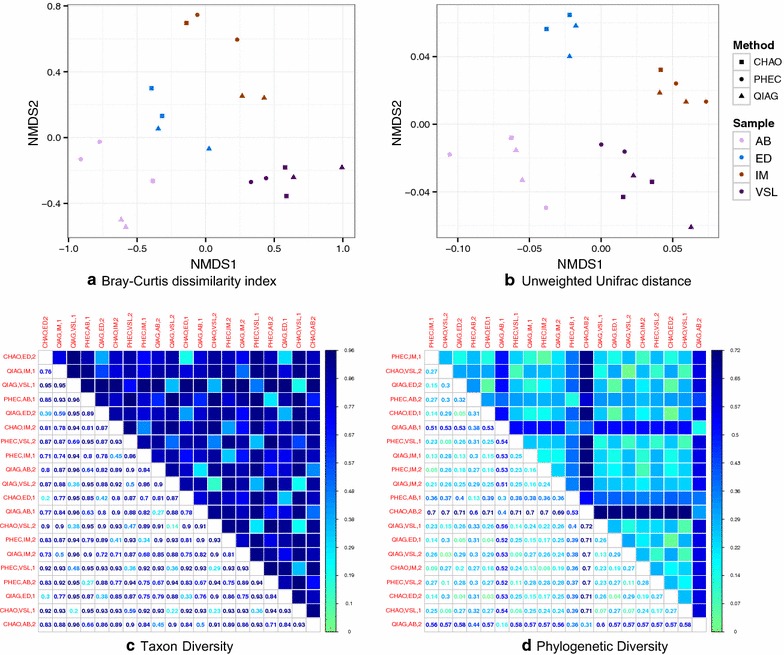
The effect of the DNA extraction methods on the compositional similarity using different β-diversity measures. **a** considers using Bray-Curtis distance based on OTU abundances alone, and **b** unweighted Unifrac distance. **c**, **d** are then the resulting beta diversity estimates using the BAT package. The lightest shade/smallest value in a given row/column represents the most similar sample in terms of community profile. ED, AB, IM correspond to the three faecal sample and VSL to the proprietary probiotic capsule ID respectively. Technical replicates represented by 1 and 2 in the sample names

For a range of α-diversity measures, a distinct pattern was not observed for different extraction methods (Fig. 3). Using different β-diversity measures for between sample similarities, on non-metric multidimensional scaling plots, a clear separation was observed between the sample types but not according to the extraction methods (Fig. 4a, b). This was the same when considering distances between samples calculated on OTU abundance counts (Bray–Curtis distance) or on their phylogenetic relatedness (unweighed Unifrac) (Fig. 4a, b). These visual cues were then confirmed using PERMANOVA (using vegan’s adonis package in R) analysis with sample type accounting for 51 % (p = 0.001) and extraction method explaining 11 % (p = 0.023) of the variation in community structure using the Bray–Curtis dissimilarity index. However, when unweighted Unifrac distance was used, the majority of the variation (R^2^ = 0.75; p = 0.001) was attributed to sample type, with extraction method becoming non-significant (R^2^ = 0.049; p = 0.114). Using a recently proposed diversity estimator (BAT package in R), the technical replicates (a measure of method reproducibility) were closer to each other for the QIAG and CHAO methods than the PHEC method (Fig. 4c, d).

**Fig. 5:**
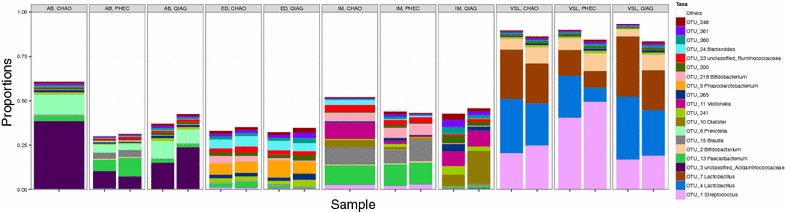
Stacked barplot of 20 most abundant OTUs from each sample along with taxonomic assignment at genus level where feasible. All sample types had distinct community signatures with different kits agreeing on community members and their ordering. Note that we have collated all other OTUs together in the “Others” category. ED, AB, IM correspond to the three faecal sample and VSL to the proprietary probiotic capsule ID respectively

**Fig. 6:**
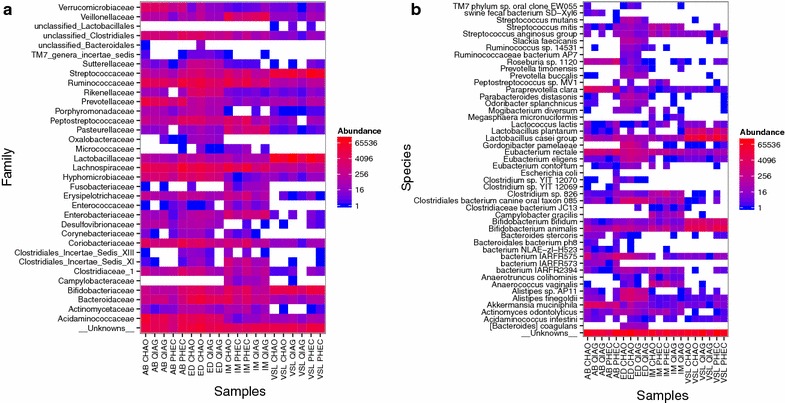
Heatmap of OTU abundances from each sample when binned at family **a** level using RDP classifier and when binned at species **b** level by blasting the sequences against NCBI database. The samples on x-axis are ordered by sample types and the colours were assigned in the log-transformed abundance scale. The OTUs were put in the “__Unknowns__” class when no taxonomic assignment was available at family or species level. ED, AB, IM correspond to the three faecal sample and VSL to the proprietary probiotic capsule ID respectively

Even though each sample had distinct community profiles, taxon membership in each of them was independent of the extraction method used (Fig. 5). Similarly, no obvious differences were observed between the three methods at taxon abundance (Fig. 6).

## Discussion

In this study we have observed that different DNA extraction methods for gut microbiota research applications lead to different results in downstream data analysis. No DNA extraction method was superior or inferior to another and the implications of their use will depend on the type of applications considered (Table 1). When the objective is to estimate the absolute concentration of the bacterial taxa in applications such as qPCR, the usage of the CHAO method will give higher values per mass of faecal material compared with the QIAG kit; a result which was merely explained by the higher double stranded DNA yield of the former method. The loss of nucleic acids in the QIAG kit is difficult to ascertain but this is likely to be associated with loss of the nucleic acids within the spin column during the purification process step; albeit the latter step might improve the quality of resulting nucleic acids. These results preclude comparison of qPCR data produced from samples extracted with different methods. The higher yield of the CHAO may also be of relevance in microbiota research with specimens of very low bacterial biomass, as in profiling of the mucosal microbiota. However, when the research objective is to study the community structure and estimate the relative abundance of taxa, using next generation sequencing of the 16S rRNA amplicon gene, no substantial differences are expected to be observed between the various DNA extraction approaches tested here.

**Table 1:** Summary of findings reported in this study

Methods	Pros	Cons
Chaotropic (CHAO)	Highest DNA yield	Most fractional loss of reads after different steps of bioinformatics pipeline
	High coverage in sequencing	Takes longest to perform
	Lowest loss of read trimming	
	Cheap	
Phenol Chloroform (PHEC)	Cheap	In the middle in terms of performance compared with the other methods
	Highest dsDNA in probiotic sample	Use toxic reagents
	Quicker than CHAO	
QIAamp DNA stool minikit (QIAG)	Highest read yield post OTUs assignment	Lowest DNA yield
	Quick	Not cheap
	Highest quality DNA	Lowest coverage
	Can be automated	

From a bioinformatics analysis perspective, an important determinant of a suitable DNA method is the high quality sequencing yield. A method which produces a high number of reads per sample, and with a high percentage of reads mapping to OTUs following bioinformatics analysis is desirable. Such a method will be more useful for statistical analysis and will be more cost-effective by decreasing the number of repeated sequencing analysis of some samples (often a library size cut off is applied to filter out samples with low abundance count). In this context, the QIAG was superior over the two other approaches.

The main limitation of this study is the small number of samples which precluded formal statistical analysis for some outcome measures. However, even with this small number of samples the majority of the results were consistent and of the same direction. Moreover, the results of this study should be interpreted with relevance to faecal specimens and the performance of these methods in other matrices (e.g. soil or plants) needs to be explored.

As research in the area of gut microbiology is moving from small scale to large multicentre international studies, a choice of practical, quick and cost effective methods will be preferred. In this instance, the CHAO method takes up to 2 days to process 15 samples at an average cost of less than £1 per sample in consumables, whereas the PHEC extraction can be completed in less than 6 h, at the same average cost as the CHAO method, and the QIAG kit will only take 3 h at an approximate cost of £4 per sample for the same number of samples and can be incorporated within automated DNA extractors.

## Conclusions

In conclusion, we have shown that there is no superior DNA method fitting all downstream approaches and the method of choice depends on the intended type of analysis, practicality and cost (Table 1). Nonetheless, we advocate towards the importance of using the same DNA extraction method when comparing group differences in a study as well as caution should be paid when the intention is to analyse biobanked samples or pool data from studies which have used different DNA extraction methods.

## Methods

### Sample collection

Faecal samples were obtained from a healthy adult, a healthy child, and a child with with Crohn’s disease who participated in ongoing research [7]. A proprietary probiotic preparation containing 8 different strains of lactic-acid bacteria, VSL#3^®^ (sigma-tau Ethifarma b.v. NL), was analysed too.

### DNA extraction methods

Extraction of 200 mg stool was carried out in duplicate for each method (technical replicates) as described below. Three different DNA extraction protocols were used: (a) a modified commercial kit (QIAamp^®^ DNA stool mini kit, Qiagen), (b) a phenol–chloroform extraction method and c) a method using a combination of chemical, enzymatic and physical steps.

### QIAamp^®^ DNA stool mini kit

The kit was applied according to the manufacturer’s instructions with modifications. These included destruction of bacterial cells with bead beating (Tungsten Carbide Beads 3 mm Cat No. 699997, QIAGEN) for 3 min at 4.5 m/s with a FastPrep-24 (MP biomedicals).

### Phenol–chloroform extraction

The phenol–chloroform extraction used is based on the protocol by Reichardt et al. [8]. This involved mechanical lysis destruction of bacterial cells with zirconium beads (0.1 mm, Biospec products) in sterile PBS, saturated acid phenol and separation of nucleic acids with chloroform/isoamylalcohol (24:1), centrifugation and precipitation of nucleic acids with isopropanol occurred in the presence of 3 M sodium chloride.

### Chaotropic method

A modified version of the protocol by Godon et al. [9] as described previously [10]. Briefly faeces were suspended in a buffer containing a salt solution and incubated for 1 h at 70 °C. Sterile silica beads (0.1 mm, Biospec products) were used for bacterial cell lysis in a FastPrep-24 bead beater (MP biomedicals). Then 15 mg Polyvinylpyrrolidone was added and the suspension was centrifuged with 15.000×*g* at 4 °C for 3 min. The supernatant was recovered, the pellet was washed with 450 μL TENP buffer, centrifuged again and washed two more times. The pooled supernatants were centrifuged with 20,000×*g* at 4 °C for 10 min. Nucleic acids were precipitated with isopropanol. Following 10 min incubation at room temperature the mixture was centrifuged with 15,000×*g* at 4 °C for 5 min and the supernatant was discarded. The pellet was resuspended in 225 μL phosphate buffer 0.1 M (pH 8) and 25 μL potassium acetate 5 M and left at 4 °C overnight. 5 μL RNAse (10 mg/mL) was added and incubated at 37 °C for 45 min. DNA was precipitated using 50 μL 3 M sodium acetate and 1 mL ice cold 100 % ethanol. After incubation at −20 °C for 1 h the DNA pellet was washed three times with 70 % ethanol, dried and stored at −20 °C in TE buffer.

### Yield and purity of isolated nucleic acids

Bacterial genomic double stranded DNA yield was measured with the Qubit^*®*^ fluorometer 2.0 using the high sensitivity assay kit (ThermoFisher, Q32851) and the purity of nucleic acids was assessed with at the absorbance ratio 260:280 (NanoDrop^*®*^ ND-1000).

### Quantitative real time PCR

The concentration of 16s rRNA gene copies of major dominant and subdominant bacterial taxon groups (*Clostridium leptum*, Clostridium *coccoides*, *Bifidobacterium genus*, *Lactobacillus*, *Escherichia coli*, *Entrerococcus*) were measured in triplicate using quantitative real-time PCR analysis on a 7500 Real-Time PCR System (Applied Biosystems) using TaqMan Gene Expression and the same primers and probes as described previously [10]. The concentration of 16S rRNA gene copy number for each sample was expressed per gram of dry faecal material taking into account any dilution factor in the concentration of template DNA in qPCR reaction. Non template controls were included in each run.

## 16S rRNA gene sequencing

16S rRNA gene sequencing was performed on the MiSeq (Illumina) platform using 2 × 250 bp paired-end reads. The V4 region was amplified using fusion Golay adaptors barcoded on the reverse strand as described previously [7]. The forward 16S rRNA primer sequence 515f (GTGCCAGCMGCCGCGGTAA) was used. The reverse primers, barcodes and adaptors were identical to those described previously [11]. Amplicons were purified with AMPure XP DNA purification beads (Beckman Coulter, Danvers, MA, USA) according to the manufacturer’s instructions, and eluted in 25 μl of proprietary elution buffer (Qiagen, 19086, UK). Amplicon concentration was quantified with use of KAPA SYBR^®^ FAST qPCR Kit (Kapa biosystems, KK4824, UK), diluted to 40 pM and spiked with 40 pM of genomic DNA to avoid base-calling issues due to low base diversity. A negative extraction control was included for each method.

### Bioinformatics

The paired end reads were trimmed and filtered using Sickle v1.200 [12] by applying a sliding window approach and trimming regions where the average base quality drops below 20. After this, we applied a 10 bp minimum length threshold to discard all shorter reads. We then used pandaseq v(2.4) [13] with a minimum overlap of 50 bp to assemble the forward and reverse reads into a single sequence spanning the entire V4 region. After obtaining the consensus sequences from each sample, we used the UPARSE (v7.0.1001) pipeline (https://bitbucket.org/umerijaz/amplimock/src) for OTU construction. In brief, we pooled reads from different samples together and added barcodes to keep an account of the samples these reads originated from. We then dereplicated the reads and sorted them by decreasing abundance and discarded singletons. In the next step, the reads were clustered based on 97 % similarity discarding reads that were shorter than 32 bp. Even though the cluster_otu command in usearch removes reads that have chimeric models built from more abundant reads, a few chimeras may be missed, especially if they have parents that are absent from the reads or are present with very low abundance. Therefore, in the next step, we used a reference-based chimera filtering step using a gold database (http://drive5.com/uchime/uchime_download.html) that is derived from the ChimeraSlayer reference database in the Broad Microbiome Utilities (http://microbiomeutil.sourceforge.net/). The original barcoded reads were matched against clean OTUs with 97 % similarity (a proxy for species level separation) to generate a total of 335 OTUs comprising all samples. The representative OTUs were then taxonomically classified against the RDP database using the standalone RDPclassifier v2.6 [14] with the default–minWords option of 5. For species level assignment, we have used NCBI Taxonomy and TAXAassign (https://github.com/umerijaz/TAXAassign). To find the phylogenetic distances between OTUs, we first multisequence aligned the OTUs against each other using mafft v7.040 [15] and then used FastTree v2.1.7 [16] on these alignments to generate an approximately-maximum-likelihood phylogenetic tree.

### Statistical analysis

Statistical analyses were performed in R using the tables and data generated as above as well as the metadata associated with the study. For community analysis (including α and β diversity analyses) we have used the vegan [17] package in R. To obtain unweighted Unifrac distances (that account for phylogenetic relatedness and are calculated using the branch lengths from the phylogenetic tree of the OTUs observed in the samples, without considering their abundances), we have used the phyloseq [18] package. Non-metric distance scaling plot (NMDS) was applied using Vegan’s metaMDS() function to visualise natural clustering in the dataset. Additionally, we have used ape [19], phangorn [20] and BAT [21] packages together to calculate the recently proposed β-diversity estimators that consider phylogeny too. The BAT package proposes three ways of estimating phylogenetic diversity (PD), building on estimators originally developed for Taxon Diversity (TD): correcting PD values based on the completeness of TD; fitting asymptotic functions to accumulation curves of PD; and adapting nonparametric estimators to PD data. The only requirement is for the phylogenetic tree to be an ultrametric tree for which we used chronos() from R’s ape package to convert our OTU tree to an ultrametric tree (after rooting the tree by applying midpoint() rooting function from the R’s phangorn package). In Fig. 4c, d, the resulting beta diversity estimates from BAT packaged are plotted using R’s corrplot to represent the quantitative estimates and then ordered using R’s hclust(). The general scripts as well as tutorials for the above analyses are available at http://userweb.eng.gla.ac.uk/umer.ijaz#bioinformatics.

## Additional files

10.1186/s13104-016-2171-7 Summary statistics of read qualities and read lengths after each step of the pipeline and before OTUs construction.
10.1186/s13104-016-2171-7 Sample raw data of read qualities and read lengths after each step of the pipeline and before OTUs construction.
