# Supplementary figures and images for: The effect of DNA extraction methodology on gut microbiota research applications
Source: BMC Res Notes. 2016 Jul 26;9:365. doi: 10.1186/s13104-016-2171-7 (PMC4960752; doi:10.1186/s13104-016-2171-7)

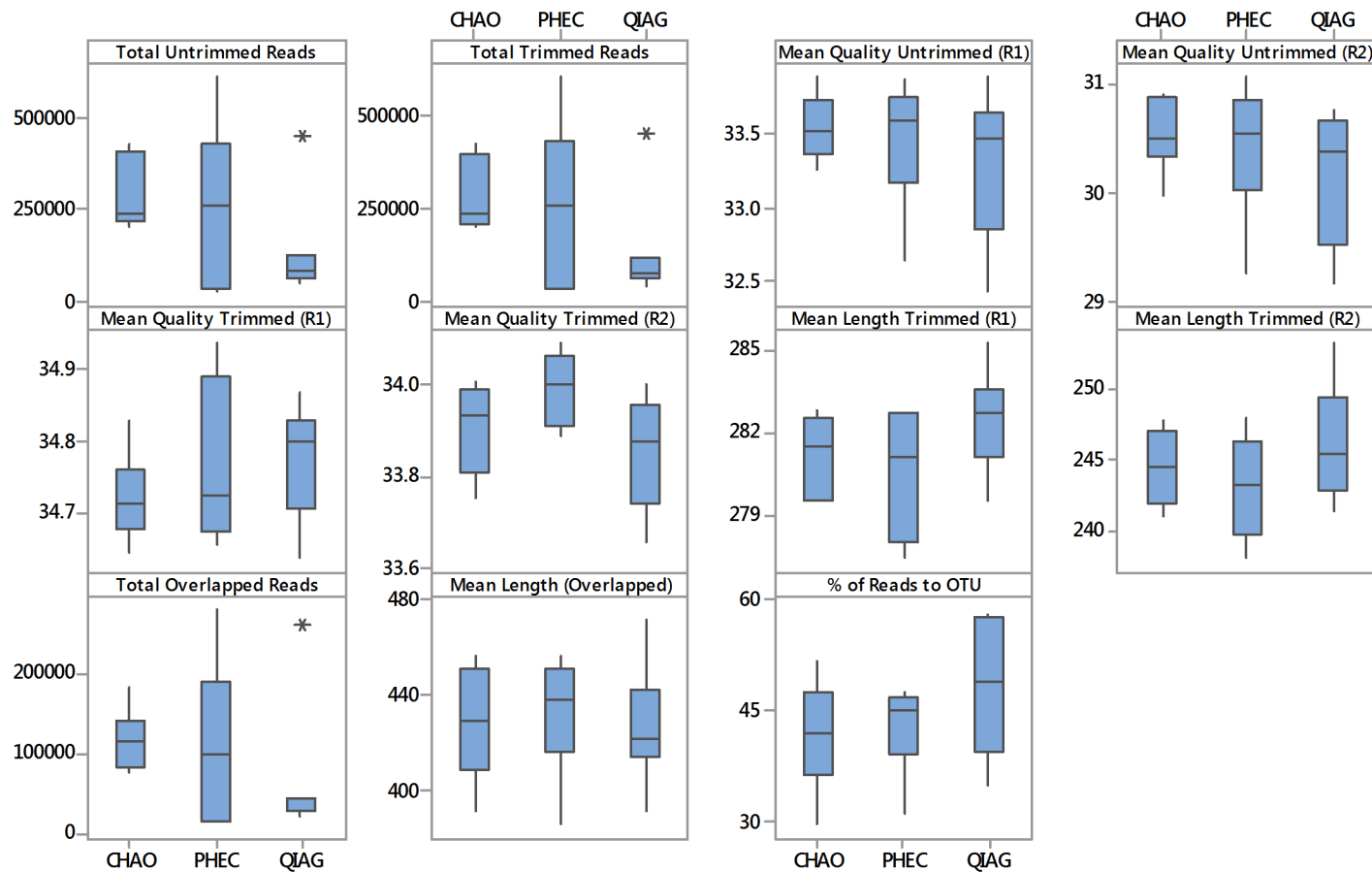

Supplement: Supplementary file 1 — 10.1186/s13104-016-2171-7 Summary statistics of read qualities and read lengths after each step of the pipeline and before OTUs construction. [file 13104_2016_2171_MOESM1_ESM.pdf]
